# Multiscale Modeling of Vascular Remodeling Induced by Wall Shear Stress

**DOI:** 10.3389/fphys.2021.808999

**Published:** 2022-01-27

**Authors:** Shiliang Chen, Hanbing Zhang, Qianwen Hou, Yu Zhang, Aike Qiao

**Affiliations:** Faculty of Environment and Life, Beijing University of Technology, Beijing, China

**Keywords:** multiscale modeling, computational fluid dynamics, agent-based model, vascular remodeling, wall shear stress

## Abstract

**Objective:**

Hemodynamics-induced low wall shear stress (WSS) is one of the critical reasons leading to vascular remodeling. However, the coupling effects of WSS and cellular kinetics have not been clearly modeled. The aim of this study was to establish a multiscale modeling approach to reveal the vascular remodeling behavior under the interaction between the macroscale of WSS loading and the microscale of cell evolution.

**Methods:**

Computational fluid dynamics (CFD) method and agent-based model (ABM), which have significantly different characteristics in temporal and spatial scales, were adopted to establish the multiscale model. The CFD method is for the second/organ scale, and the ABM is for the month/cell scale. The CFD method was used to simulate blood flow in a vessel and obtain the WSS in a vessel cross-section. The simulations of the smooth muscle cell (SMC) proliferation/apoptosis and extracellular matrix (ECM) generation/degradation in a vessel cross-section were performed by using ABM. During the simulation of the vascular remodeling procedure, the damage index of the SMC and ECM was defined as deviation from the obtained WSS. The damage index decreased gradually to mimic the recovery of WSS-induced vessel damage.

**Results:**

(1) The significant wall thickening region was consistent with the low WSS region. (2) There was no evident change of wall thickness in the normal WSS region. (3) When the damage index approached to 0, the amount and distribution of SMCs and ECM achieved a stable state, and the vessel reached vascular homeostasis.

**Conclusion:**

The established multiscale model can be used to simulate the vascular remodeling behavior over time under various WSS conditions.

## Introduction

Vascular remodeling is a process that the vessel changes its structure and function to adapt to the environmental alterations. This complex process involves endothelial hyperplasia, vascular smooth muscle cell (VSMC) proliferation/apoptosis, and extracellular matrix (ECM) generation/degradation. Vascular remodeling is a common pathology of various vascular disorders, such as atherosclerosis, bypass graft failure, in-stent restenosis, and arteriovenous fistula failure (Browne et al., 2015). It is affected by a variety of internal and external factors, for example, biology, chemistry, and physics, among which hemodynamics plays a particularly important role in vascular remodeling. Hemodynamics-induced low wall shear stress (WSS) is one of the critical reasons leading to vascular remodeling (Chatzizisis et al., 2008; Yu et al., 2015; Luong et al., 2016). WSS is a tangential friction force that blood flow exerts on the vessel wall. The pulsatility of blood flow, the rheological properties of blood flow, and the geometry of blood vessel together determine WSS, which is characterized by direction and magnitude (Chatzizisis et al., 2007). In the geometrically irregular region where disturbed flow occurs, it will produce low or oscillating WSS. The cutoff point of low WSS, physiological WSS, and high WSS varies with species and blood vessel types. Low WSS usually refers to WSS less than 1 Pa (Malek et al., 1999; Wentzel et al., 2012; Li et al., 2014; Wang et al., 2016).

Endothelial cell (EC) has WSS receptors, which can sense the changes of WSS and transduce them into biochemical signals (Baeyens et al., 2015). When VSMC receives biochemical signals, VSMC will produce vasoactive substances to act on VSMC and ECM, which leads to changes in the morphological structure of the vessel wall, ultimately adapts to mechanical environments. Therefore, we call vascular remodeling is a mechanochemical-biological process. This process involves the effect of a variety of vasoactive substances, such as nitric oxide (NO), platelet-derived growth factor, and matrix metalloproteinases (MMP). NO synthesized by NO synthase in EC has strong anti-inflammatory, antiapoptotic, antimitotic, and antithrombotic effects and can protect blood vessels. The activity of NO synthase is regulated by normal WSS, and the protein level of endothelial NO synthase is lower in low WSS, which weakens the protective effect on blood vessels (Yamamoto and David, 2010; Liu and Chen, 2020). Low WSS promotes the expression of platelet-derived growth factor, vascular endothelial growth factor, and pro-inflammatory cytokines, inhibits the expression of transforming growth factor, which contributes to the phenomena of overexpression of growth-promoting factor and the underexpression of growth-inhibiting factor, and ultimately promotes the phenotype transformation, migration, differentiation, and proliferation of VSMC (Grainger, 2004). The gene expression and activity of MMP are upregulated by low WSS, especially MMP-2 and MMP-9 (Cheng et al., 2006), which are the main proteases related to ECM degradation. ECM degradation will promote VSMC migration. In addition to promoting ECM degradation, low WSS also reduces ECM synthesis. Interferon is a pro-inflammatory cytokine produced by activated T lymphocytes, which responds to low WSS and is an effective inhibitor of ECM synthesis (Tousoulis et al., 2006).

In the past three decades, the computational simulation method has emerged as a powerful tool to investigate biological phenomena, for example, studying causes, mechanisms, and therapeutic approaches of cardiovascular disease. By using the computational fluid dynamics (CFD) method, we could simulate blood flow in a special location of the cardiovascular system and have new insights into hemodynamics. CFD also shows hemodynamic parameters from the flow field, such as velocity, pressure, and WSS. The methods for vascular remodeling simulations can be divided into two types, namely, continuum models and discrete models (Garbey et al., 2015; Escuer et al., 2019). Continuum models usually use partial differential equations to describe the state changes of a system. One of the advantages of continuum models is that partial differential equations provide a clear mathematical relationship between the components in the system. In addition, the continuum models can be used to model all levels of a system. However, the continuum models do not reflect the state of discrete individuals and the interaction between discrete individuals in the system, while the discrete models such as the agent-based model (ABM) simulate each individual behavior and interactions among them. Therefore, the difference between the two types is that the former focuses on the overall state of the system, while the latter focuses on the interaction between individuals in the system. ABM is a discrete time, discrete event, rule-based modeling method. ABM includes various types, for example, lattice-based, lattice-free, Cellular Potts, lattice-gas, and subcellular element modeling methods (Wang et al., 2015). In ABM, each element usually represents an agent. Agents perform corresponding behaviors in the simulation process according to defined rules, including their own independent behaviors and interactions with other agents. Based on the characteristics of ABM, ABM is widely used in the simulation of biological phenomena, such as tumor microenvironment (Norton et al., 2019), bacterial activity in the soil habitat (Borer et al., 2019), and the transmission of new coronavirus disease 2019 (COVID-19) (Rockett et al., 2020). In addition, ABM is also widely used to simulate the activities of EC and VSMC. These studies mainly focus on in-stent restenosis (Boyle et al., 2010; Thorne et al., 2011; Tahir et al., 2014; Zahedmanesh et al., 2014; Zun et al., 2017; Keshavarzian et al., 2018, 2019; Li et al., 2019). For example, Zahedmanesh et al. (2014) investigated the role of MMP and ECM changes during in-stent restenosis by using a multiscale mechanobiological model. This multiscale mechanobiological model is 2D. Based on the 2D model, Zun et al. (2017) developed a 3D multiscale model of in-stent restenosis to study the effects of stent deployment depth, stent width, and reendothelization speed on the process of restenosis. A fully coupled ABM-finite element analysis (ABM-FEA) framework of in-stent restenosis was developed by Li et al. (2019) to overcome the limitation that the traditional ABM-FEA framework was often partially coupled. The abovementioned studies are based on the combination of ABM and FEA, which is called the multiscale simulation method. The FEA is for the second/organ scale, and the ABM is for the month/cell scale.

As mentioned above, WSS is one of the reasons that lead to vascular remodeling, which is based on experiments and statistics. However, the coupling effects of WSS and cellular kinetics have not been clearly modeled. The aim of this study was to establish a multiscale modeling approach to reveal the vascular remodeling behavior under the interaction between the macroscale of WSS loading and the microscale of cell evolution. CFD method and ABM, which have significantly different characteristics in temporal and spatial scales, were adopted to establish the multiscale model. The CFD method was used to simulate blood flow in a vessel and obtain the WSS in a vessel cross-section. The simulations of VSMC proliferation/apoptosis and ECM generation/degradation in a vessel cross-section were performed by using ABM.

## Materials and Methods

### Model Overview

The multiscale model of vascular remodeling contained two parts: the CFD model, which simulated blood flow in a vessel to obtain the WSS in a vessel cross-section, and the ABM, which simulated VSMC proliferation/apoptosis and ECM generation/degradation in a vessel cross-section. These two parts were coupled by a parameter named damage index, which was defined as deviation from the obtained WSS. The damage index was used to determine the probability of VSMC proliferation/apoptosis and ECM generation/degradation.

### Computational Fluid Dynamics Model

**FIGURE 1:**
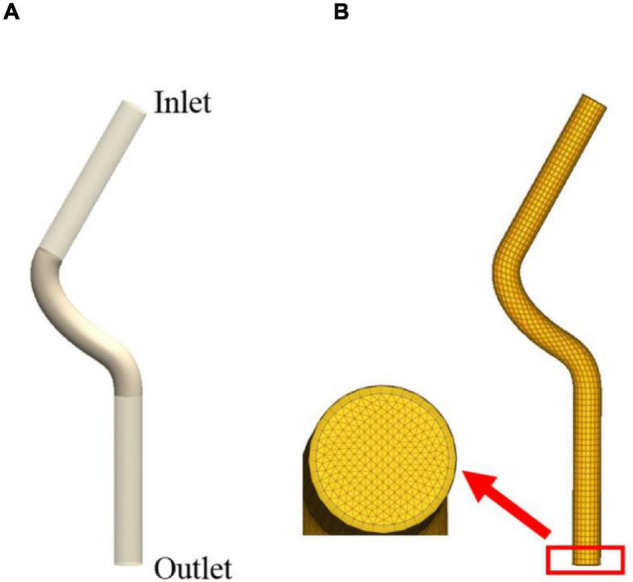
The 3D geometry of the proximal left anterior descending branch **(A)** and meshing result **(B)**.

The vascular remodeling of the proximal left anterior descending branch was investigated in this study to validate the proposed method. The 3D geometry of the proximal left anterior descending branch was a simplified conceptual model. It was reconstructed based on the actual radius and centerline which we got from the CT data, as illustrated in Figure 1A. The proximal left anterior descending branch is the middle part, and we extended both inlet and outlet to obtain a fully developed flow. The proximal left anterior descending branch has a radius of 1.535 mm and a length of 19.253 mm. Meshing and CFD simulations were carried out by using commercial software. The calculation domain was described by a nonstructural grid as shown in Figure 1B. Blood was considered as an incompressible Newtonian fluid with a density of 1,060 kg/m^3^ and a viscosity of 0.0035 Pa⋅s. Laminar flow at a speed of 50 ml/min was applied (Theodorakakos et al., 2008). The blood was set to be laminar flow because the Reynolds number was < 2,000 when the flow rate was set to 50 ml/min. The WSS was obtained by using steady CFD analysis. The wall of the model was assumed to be rigid with a nonslip boundary condition, and zero pressure was set at the outlet. The WSS of each element in a vessel cross-section, which would be utilized as the input for ABM simulations, was recorded at the end of the CFD simulation.

### Agent-Based Model

**FIGURE 2:**
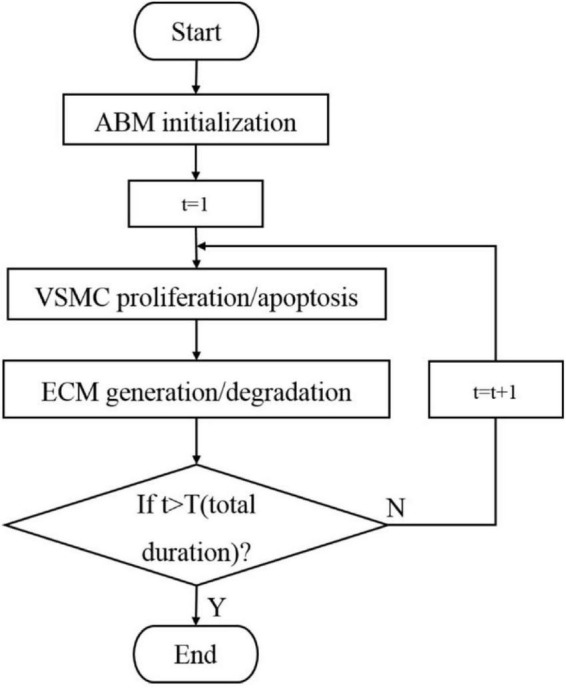
Flow diagram of an agent-based model (ABM) in the vessel cross-section.

**FIGURE 3:**
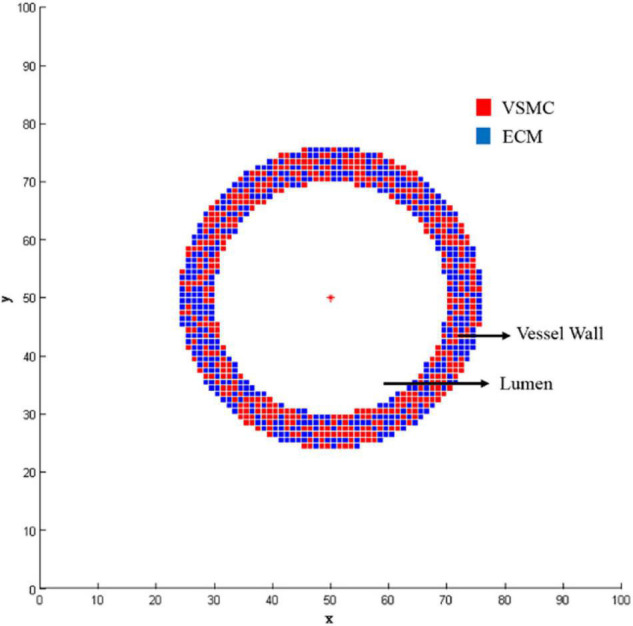
Geometry initialization.

An ABM was developed to simulate VSMC proliferation/apoptosis and ECM generation/degradation in a vessel cross-section. The ABM of a vessel cross-section was developed in MATLAB (R2014a). Figure 2 shows the flow diagram of ABM. As initial setup, the initialization of ABM included three parts, namely, geometry initialization, time initialization, and hemodynamic initialization. The simulation area was a 2D domain, which was discretized uniformly into 100 × 100 grids. The proximal left anterior descending branch was a simplified conceptual model, so the cross-section of the vessel is an ideal circle. We set a circular ring where the center was (50, 50) and radius was between 20 and 26 as the boundary of the vessel wall. The center of the lumen was set to be (50, 50), and its radius was 20. The proximal left anterior descending branch was selected as the vascular model, where the actual radius of the proximal left anterior descending branch was 1.535 mm, and the corresponding thickness of the vessel wall was 460.5 μm, which was consistent with the literature (Waller, 1989). Therefore, the rationality of the radius and wall thickness in ABM is acceptable. The ratio of VSMC/ECM was 1. The initial cellular pattern in a vessel cross-section was random according to the published literature (Hwang et al., 2013; Garbey et al., 2015; Casarin et al., 2018). Figure 3 shows the vessel cross-section after geometry initialization. Vascular remodeling was a process that evolves over time, so we set a time clock for VSMC and ECM agents. The cycle of VSMC was 12 h, and that of ECM was 4 h (Garbey et al., 2015). Each VSMC and ECM agent had a random age between 0 and cycle. As simulation began, the age increased according to the time step of 1 h. A function was used to define the damage index of VSMC and ECM as deviation from the obtained WSS as followed:


(1) D={WSS0−WSSWSS0    WSS<WSS0        0       WSS>WSS0

where WSS_0_ is a threshold for damage. The threshold for damage was 1 Pa, since low WSS usually refers to WSS less than 1 Pa (Malek et al., 1999; Wentzel et al., 2012; Li et al., 2014; Wang et al., 2016). That is, when WSS is greater than or equal to 1 Pa, its damage index is 0. When WSS is close to 0 Pa, its damage index is the largest, which is 1. When WSS is between 0 and 1, its damage index is linearly distributed (Boland et al., 2019).

The agent in the innermost layer of the vessel wall got the damage index, and then, the damage index of agents in the other layer was defined by the inner damage index and attenuation coefficient according to distance. That is, for an agent in the outer layer having the closest agent in inner layer, the damage index of the former one was defined as the latter one multiplied by the attenuation coefficient. After hemodynamic initialization, each agent in the vessel wall got a damage index. It showed that the lower the WSS, the greater the damage index. The damage index decreased gradually from the inner wall to the outer wall. During the vascular remodeling process, the lumen area decreased, which increased the WSS. Therefore, in each time step, the damage index was multiplied by a constant *e*^−0.0075^ to replicate the WSS recovery. The damage index was used to calculate the probability induced by WSS, the functions of VSMC division probability, VSMC apoptosis probability, ECM generation probability, and ECM degradation probability were as follows:


(2) $${\text{p}}_{\mathrm{div}-\mathrm{wss}}=\alpha_{\text{div}}\,\text{×D}$$

(3) $${\text{p}}_{\mathrm{apop}-\mathrm{wss}}=\alpha_{\text{apop}}\,\text{×D}$$

(4) $${\text{p}}_{\mathrm{gen}-\mathrm{wss}}=\alpha_{\text{gen}}\,\text{×D}$$

(5) $${\text{p}}_{\deg-\mathrm{wss}}=\alpha_{\text{deg}}\,\text{×D}$$

where α_div_, α_apop_, α_gen_, and α_deg_ are the parameters used to modify the probability.

Vascular remodeling is a mechanochemical-biological process. We considered the interaction between WSS, biochemical factors, VSMC, and ECM. These biochemical factors included Endothelin (ET), NO, and MMP-9. The mathematical relationships between WSS, biochemical factor content, VSMC, and ECM were established according to the literature (Guo and Kassab, 2009; Thorne et al., 2011; Nirala and Gohil, 2015). ET can be synthesized by a variety of cells, such as EC, VSMC, glial cell, and cardiomyocyte, mainly EC. ET can promote the proliferation and migration of VSMC. The mathematical relationship of WSS, EC, and ET was as follows (Thorne et al., 2011):


(6) $${\text{C}}_{\text{ET}}=M\,\left(\delta +\alpha \,\left(1-{\text{e}}^{-k\,\tau^{n}}\right)\right)$$

where C_ET_ represents the ET synthesized by a single EC per hour, the unit is pg/cell/h, τ represents WSS, the unit is Pa, and *M*=8×10^−4^pg/cell/h, δ=0.6, α=0.4, *k*=3.63, *n* = 1.68.

The NO in the vessel is mainly synthesized by EC, which mainly acts on VSMC and inhibits VSMC proliferation and migration. The mathematical relationship of WSS, EC, and NO was as follows (Guo and Kassab, 2009):


(7) $${\text{C}}_{\text{NO}}=a\,\tau^{4}+b\,\tau^{3}+c\,\tau^{2}+d\,\tau +e$$

where C_NO_ represents the NO synthesized by a single EC per hour, the unit is pg/cell/h, τ represents WSS, the unit is Pa, and *a*=4.365×10^−7^,*b*=−9.399×10^−7^, *c*=6.348×10^−7^, *d*=−9.939×10^−8^, *e*=9.333×10^−9^.

The main physiological role of MMPs is to degrade the ECM. More than 20 kinds of MMPs have been discovered so far. MMP-9 is one of them, which belongs to gelatinase and has a wide range of substrates. The mathematical relationship of WSS, EC, and MMP-9 was as follows (Nirala and Gohil, 2015):


(8) $${\text{C}}_{\mathrm{MMP}-9}=\beta \,\tau +\gamma$$

where C_*MMP*−9_ represents the MMP-9 synthesized by a single EC per hour, the unit is pg/cell/h, τ represents WSS, the unit is Pa, and β=4.939×10^−8^,γ=2.218×10^−7^.

After obtaining the content of ET, NO, and MMP-9 at a certain time, the mathematical relationship between the content of biochemical factors and the interaction of VSMC and ECM should be established. Substituting the content of biochemical factors into the following formulas, the content of biochemical factors could be converted into probability:


(9) pET=16.67-1.45×10-9⁢CET*+8×104

(10) pNO=16.67-1.45×10-9⁢CNO*+8×104

(11) pMMP-9=16.67-1.45×10-9⁢CMMP-9*+8×104

where C_ET_, C_NO_, C_*MMP*−9_ represent the content of ET, NO, and MMP-9, respectively.

The p_div−wss_, p_apop−wss_, p_gen−wss_, and p_deg−wss_ were calculated based on the damage index, and the probability of the biochemical factor was superimposed on the abovementioned probability. Thus, the VSMC division probability P_div_, VSMC apoptosis probability P_apop_, ECM generation probability P_gen_, and ECM degradation probability P_deg_ could be obtained. Considering that ET promotes the proliferation of VSMC and NO inhibits the proliferation of VSMC, the probability of VSMC division was modified as follows: P_div_=p_div−wss_ + p_ET_−p_NO_. The probability of VSMC apoptosis was not modified, P_apop_=p_apop−wss_, because the concepts of promoting proliferation and inhibiting proliferation have nothing to do with apoptosis. Because MMP-9 can promote ECM degradation, the ECM degradation probability was modified as follows: P_deg_=p_deg−wss_ + p_MMP−9_, and the probability of ECM generation was not modified, P_gen_=p_gen−wss_.

**FIGURE 4:**
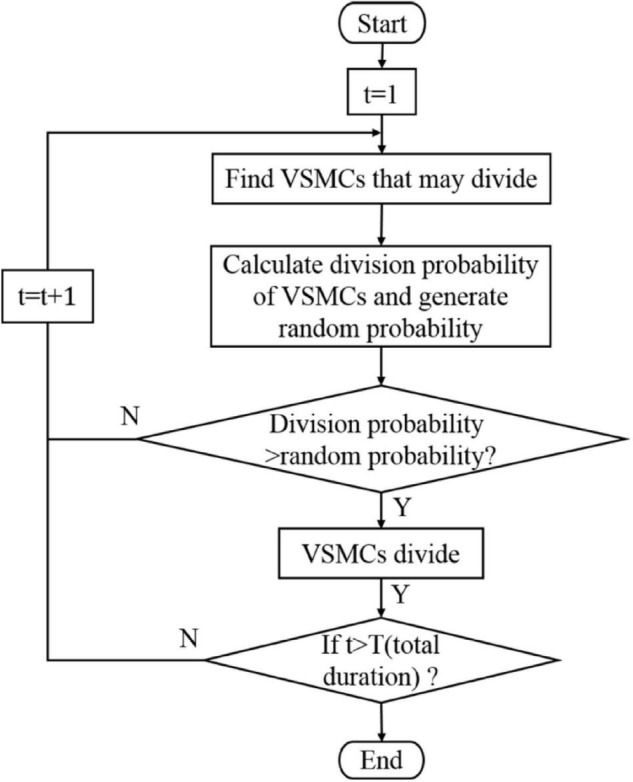
Flow diagram of the vascular smooth muscle cell (VSMC) division.

Figure 4 shows the flowchart of the VSMC division. Simulating VSMC division mainly includes the following steps: (1) Find VSMC that may divide: select the VSMC that may divide according to the age, when the age of VSMC is an integral multiple of 12, the VSMC is likely to divide, (2) Determine whether the VSMC that may divide will divide: a random probability P_div_rand_ between 0 and 1 is generated for the VSMC that may divide. The random probability P_div_rand_ is compared with the probability division for VSMC. If the probability is larger than the random probability, then division for VSMC happened, otherwise it does not happen. The age of VSMC that has not been divided is increased by 1 h, and the abovementioned steps are repeated in the next time step. VSMC apoptosis and ECM generation/degradation are the same as VSMC division. The age of the new VSMC and ECM agents was 1, and the damage value was equal to the original agent.

In the simulation, we mimicked the process of positive and negative remodeling. Positive remodeling usually occurs at the early stage of vascular remodeling, which delays the degree of vascular stenosis. Only when the plaque burden exceeds 40%, the blood flow will be affected (Glagov et al., 1987). In the late stage of the vascular remodeling, as the vascular stenosis progresses, along with calcium salt deposition and fibrosis in the plaque, it causes further narrowing of the lumen, that is, negative remodeling. During the simulation process, when the plaque burden was less than 40%, the growth direction was outward of the wall; when the plaque burden was greater than 40%, the growth direction was inward of the wall.

## Results

**FIGURE 5:**
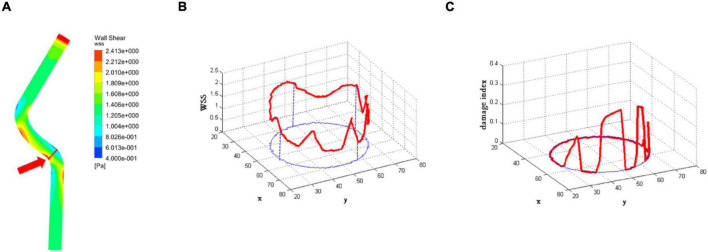
The results of the computational fluid dynamics (CFD) model. **(A)** The wall shear stress (WSS) of the proximal left anterior descending branch. The location indicated by the red arrow is the selected cross-section. **(B)** The WSS of the selected cross-section. The red curve in the figure is drawn based on WSS, and the blue curve is the projection of WSS data in the X-Y plane. **(C)** The damage index of the selected cross-section. The red curve in the figure is drawn based on the damage index, and the blue curve is the projection of the damage index in the X-Y plane.

The first part of the multiscale model of vascular remodeling was the CFD model, which simulated blood flow in a vessel to obtain the WSS in a vessel cross-section. The WSS of the proximal left anterior descending branch was obtained by the CFD method, as shown in Figure 5A. A low WSS region was observed, and we randomly selected a cross-section in this region. The location indicated by the red arrow was the selected cross-section. Figure 5B shows the WSS of the selected cross-section, the red curve in the figure is drawn based on WSS, and the blue curve is the projection of the WSS data in the X-Y plane. Furthermore, WSS was converted into WSS damage index according to the damage index equation (1), as shown in Figure 5C. The red curve in the figure is drawn based on the damage index, and the blue curve is the projection of the damage index in the X-Y plane. When WSS was greater than the threshold WSS_0_, the damage index is 0; when the WSS was less than the threshold WSS_0_, the damage index showed the trend that the smaller the WSS was, the greater the damage index was.

**FIGURE 6:**
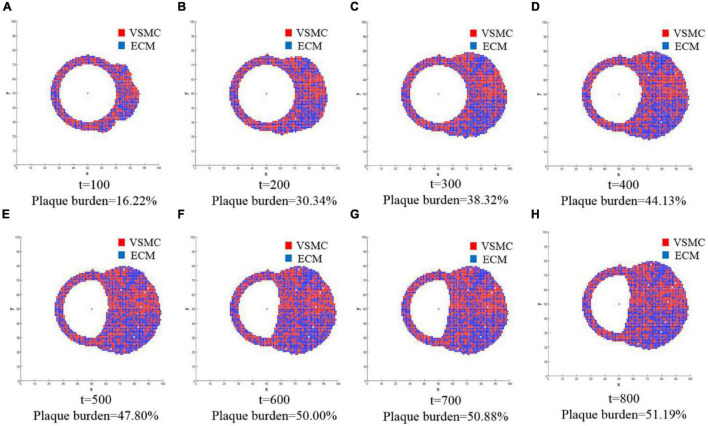
The changes of geometry in the vessel cross-section over time were obtained from a single run of the ABM. A total of 800 ticks are simulated, and the results are extracted every 100 ticks. The results from *t* = 100 to *t* = 800 are shown in **(A–H)**, respectively. Lumen is represented in white, and the vessel wall consists of VSMC and extracellular matrix (ECM): VSMC in red and ECM in blue. The figure not only shows the change of geometry but also the change of plaque burden.

The second part of the multiscale model of vascular remodeling was the ABM, which simulated VSMC proliferation/apoptosis and ECM generation/degradation in a vessel cross-section. Since VSMC proliferation/apoptosis and ECM generation/degradation were probabilistic events, the simulation results of each run of the ABM were random. Figure 6 compares the vessel cross-section geometry over time obtained from a single run of the ABM. A total of 800 ticks were simulated, and the results were extracted every 100 ticks. The results from *t* = 100 to *t* = 800 are shown in Figures 6A–H, respectively. Lumen is represented in white, VSMC in red, and ECM in blue. In terms of geometry, the results of the cross-sections we obtained were consistent with the clinical histologic images (Varnava et al., 2002; Kroner et al., 2011). As shown in the figure, the vessel wall underwent positive remodeling from *t* = 1 to *t* = 400, the lumen area remained unchanged, and the vessel wall thickened outward. After *t* = 400, the vessel wall thickened inward, and lumen began to narrow, which was called negative remodeling. Comparing the geometry of a vessel cross-section with the WSS results, we can observe that the significant wall thickening region was consistent with the low WSS region. There was no evident change of wall thickness in the normal WSS region. The figure shows not only the change of geometry but also the change of plaque burden. The plaque burden can be calculated from the cross-section of vessels, and it increased gradually and ultimately reaches 51.19%. It can be observed that the change rate of plaque burden changed from fast to slow. This was because the damage index decreases continuously during the simulation, that is, the probability of VSMC proliferation/apoptosis and ECM degradation/generation decreased. At *t* = 800, the damage index approached to 0, the quantity and distribution of VSMC and ECM achieved a stable state, and the vessel reached vascular homeostasis.

**FIGURE 7:**
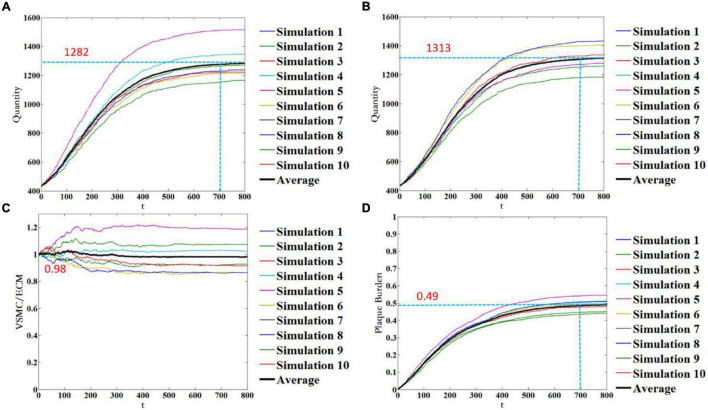
Temporal evolution of the quantity of VSMC **(A)**, the quantity of ECM **(B)**, the ratio of VSMC/ECM **(C)**, and plaque burden **(D)**. Notably, 10 stochastic simulations of the ABM are shown in color, and the mean trend is shown in black color.

As mentioned earlier, the simulation results of each run of the ABM were random. This was not only because VSMC proliferation/apoptosis and ECM degradation/generation were the probabilistic events, but also because the spatial distribution and age of VSMC and ECM were random during geometry initialization and time initialization. To minimize the influence of the probabilistic events and the random setting of initial conditions, more stochastic simulations need to be performed. Figure 7 shows 10 stochastic simulations of the ABM and mean trend. Figures 7A–D represents the quantity of VSMC, the quantity of ECM, the ratio of VSMC/ECM, and plaque burden, respectively. The quantity of VSMC increased according to the trend of the logic curve and gradually tended to be stable when *t* = 700. Under this WSS condition, the quantity of VSMC fluctuated around 1,282 (Figure 7A). The quantity of ECM also increased according to the trend of the logic curve and gradually tended to be stable when *t* = 700. Under the same WSS condition, the quantity of ECM fluctuated around 1,313 (Figure 7B). As shown in Figure 7C, the results of the 10 stochastic simulations showed that the ratio of VSMC/ECM was symmetrically distributed, and after reaching stability, the ratio of VSMC/ECM was slightly lower than the initial value and was 0.98. This was because the quantity of VSMC was slightly less than that of ECM. The plaque burden had the same trend as the quantity of VSMC and the quantity of ECM. The mean trend of plaque burden under this WSS condition was finally stable at 49%. Thus, although the results of each stochastic simulation were different, the trend of each simulation result remained the same. It also showed that the spatial distribution and age of VSMC and ECM were random during geometry initialization, and time initialization had less effect on the simulation results.

## Discussion

There is growing evidence that the hemodynamics-induced low WSS is one of the critical reasons leading to vascular remodeling (Roux et al., 2020; Mahmoudi et al., 2021). However, the coupling effects of WSS and cellular kinetics have not been clearly modeled. We established a multiscale modeling approach to reveal the vascular remodeling behavior under the interaction between the macroscale of WSS loading and the microscale of cell evolution. In the model of vascular remodeling induced by WSS, the simulations of VSMC proliferation/apoptosis and ECM generation/degradation in a vessel cross-section were performed.

The simulation results demonstrated that the multiscale model could simulate the mechanical-biological behavior between WSS, VSMC, and ECM. The significant wall thickening region was consistent with the low WSS region. There was no evident change of wall thickness in the normal WSS region. This phenomenon is consistent with the conclusion in the literature that low WSS induces vascular remodeling (Yu et al., 2015; Luong et al., 2016; Roux et al., 2020; Mahmoudi et al., 2021). Positive remodeling and negative remodeling were considered in the model of this study. It was observed that when the plaque burden was less than 40%, the vessel wall grew outward, and when the plaque burden was greater than 40%, the vessel wall grew inward, causing lumen stenosis. This phenomenon has been consistent with the previous study (Bhui and Hayenga, 2017). By comparing the results of multiple ABM simulations, it was found that although the results of each simulation were different, the trend was the same, which showed that some randomly distributed parameters in the simulation process have less influence on the results, and the main factor affecting the results was WSS. The WSS was utilized as the input for ABM simulations. Most studies have shown that low WSS is one of the reasons for vascular remodeling. Some studies suggest that vascular remodeling is related to WSS gradient (WSSG), oscillatory shear index (OSI), and relative residence time (RRT) (Gori and Boghi, 2011; Ameenuddin and Anand, 2020). WSSG, OSI, and RRT are the hemodynamic parameters derived from WSS. These parameters can provide further insight into the relationship between WSS and vascular remodeling. In this study, we only considered the effect of low WSS and did not consider the effects of high WSSG, high OSI, and high RRT. The next step of the study can be based on the other three factors to explore the differences in vascular remodeling under the action of these three factors.

Some studies have used the multiscale model to study in-stent restenosis, using Von Mises stress as input to study the effect of Von Mises stress on VSMC. Our model mainly takes WSS as input from the perspective of hemodynamics. Some models in the previous research did not consider ECM, which resulted in the simulation results being slightly smaller than the experimental data (Zun et al., 2017; Li et al., 2019). Aware of this, we considered the influence of ECM in our model.

There are several model parameters that can be adjusted to affect the dynamics of vascular remodeling. First, the degree of low WSS may be a key determinant of the individual natural history of vascular remodeling. As mentioned above, the cutoff point of low WSS, physiological WSS, and high WSS varies with species and blood vessel types. The threshold of low WSS determines the damage index and further affects the probability. Second, the periods of VSMC agent and ECM agent adopt different values in different studies, and we chose a value suitable for our model (Garbey et al., 2015; Zun et al., 2017; Corti et al., 2020). The best parameters in the model are expected to be verified by the experimental data.

## Limitations

Although the multiscale model can be used to reveal the vascular remodeling behavior under the interaction between the macroscale of WSS loading and the microscale of cell evolution, this model still has some limitations. The CFD model and boundary conditions were simplified in this study. The patient-specific CFD model should be reconstructed, and boundary conditions should be obtained to ensure the accuracy of the CFD simulation results. In addition, the vessel wall was assumed to be a rigid wall, and the interaction between blood flow and the wall was not considered, which may have an impact on the calculated results. In the future study, the fluid-structure interaction methods should be considered. During geometry initialization, we simplified the vessel cross-section by using the ideal circle. Real vessel cross-section should be used in the future study to make the model more accurate. WSS updating plays an important role in coupling the CFD and ABM. In this study, we updated WSS by using some *a priori* functions rather than using the CFD model. This approach only described a change trend and did not realize the real WSS update, which leads to the present model being partially coupled. In the future study, we could select multiple cross-sections of the blood vessel for ABM. Then, a new 3D blood model can be reconstructed by the multiple ABM results. New WSS could be obtained as input after CFD simulation. We look forward to make our model fully coupled by using this method. In our multiscale model, only the simulations of VSMC proliferation/apoptosis and ECM generation/degradation were considered. There is a phenotypic transition of VSMC during vascular remodeling, and our model does not distinguish the phenotype of VSMC. Other factors such as leaky endothelial barrier, lipid deposition, and inflammatory response were excluded in this model, and these factors should be evolved during the next study. The role of three biochemical factors was added to the model, and the content of biochemical factors was used as a function of the number of EC and WSS. This method obviously simplifies the change process of the content of biochemical factor. It is possible to further improve the description of the changes and effects of biochemical factors by establishing convection-diffusion-reaction equations. In the future study, we hope to improve the model, carry out animal experiments, and verify the model by using the results of animal experiments.

## Conclusion

With the usage of CFD and ABM, we were able to establish a multiscale modeling approach to reveal the vascular remodeling behavior under the interaction between the macroscale of WSS loading and the microscale of cell evolution. As expected, the thickening area of the vascular wall corresponded to the magnitude of WSS, showing that the lower the WSS, the easier the thickening of the vascular wall. The established multiscale model could be used to simulate the vascular remodeling behavior over time under various WSS conditions.

## Data Availability Statement

The original contributions presented in the study are included in the article/supplementary material, further inquiries can be directed to the corresponding author.

## Author Contributions

SC was responsible for modeling, simulation, data analysis, and manuscript preparation. HZ was responsible for language modification. QH assisted in the design of numerical simulation. YZ was responsible for language modification. AQ was responsible for supervision. All authors contributed to the article and approved the submitted version.

## Conflict of Interest

The authors declare that the research was conducted in the absence of any commercial or financial relationships that could be construed as a potential conflict of interest.

## Publisher’s Note

All claims expressed in this article are solely those of the authors and do not necessarily represent those of their affiliated organizations, or those of the publisher, the editors and the reviewers. Any product that may be evaluated in this article, or claim that may be made by its manufacturer, is not guaranteed or endorsed by the publisher.
